# A 6-month depot formulation of leuprolide acetate is safe and effective in daily clinical practice: a non-interventional prospective study in 1273 patients

**DOI:** 10.1186/1471-2490-11-15

**Published:** 2011-07-29

**Authors:** Ulf W Tunn

**Affiliations:** 1 Urological Clinic Facharztzentrum Klinikum Offenbach, Starkenburgring 50, 63069 Offenbach/Main, Germany

## Abstract

**Background:**

Testosterone stimulates growth in many prostate tumours. The established GnRH analogue leuprolide acetate is incorporated in a novel biodegradable polymer matrix (Atrigel^® ^delivery system), that can be administered to reduce testosterone levels in men with advanced hormone-dependent prostate cancer. This novel formulation is available as a 1-, 3- and most recently 6-month depot (Eligard^® ^45 mg). The latter was shown to lower and maintain safe and effective serum testosterone suppression in a clinical study.

**Methods:**

A non-interventional study to confirm the efficacy and safety of 6-monthly leuprolide acetate (Eligard^® ^45 mg) in routine urological practice was performed in Germany. Data were obtained from 1273 patients under the care of 634 urologists, and were analysed descriptively. Concentrations of PSA and serum testosterone were documented at the baseline visit and at 6 and 12 months following 6-monthly leuprolide acetate. The participating physicians were also asked to assess the efficacy, tolerabilty and handling of 6-monthly leuprolide acetate.

**Results:**

Serum concentrations of PSA and testosterone were decreased substantially within 6 months of initial 6-monthly leuprolide acetate administration. At 12 months, median reductions of 96% (to 0.5 ng/ml) in PSA, and 90% (to 8.9 ng/dl) in serum testosterone, were observed. Further PSA and serum testosterone decreases were also observed in a subpopulation of patients who switched to 6-monthly leuprolide acetate from other GnRH analogues. Physicians rated 6-monthly leuprolide acetate as easy to use, and patients reported good tolerability. Adverse events occurred in 9% of patients; the majority were not serious. In particular, low rates of hot flushes were reported.

**Conclusions:**

This non-interventional study showed that the reliable reduction of PSA and testosterone levels demonstrated in previous clinical studies of twice-yearly leuprolide acetate can also be achieved in routine clinical practice. This study also confirmed good tolerability of 6-monthly leuprolide acetate in routine clinical use and received positive appraisal from physicians.

## Background

According to the Robert-Koch Institute (RKI) and the Association of Population-based Cancer Registries in Germany (GEKID), there are an estimated 60,000 new cases of prostate cancer in Germany each year. Prostate cancer is the most common malignancy in men, accounting for 25% of all new cases of cancer [1]. Testosterone stimulates the growth of most prostate tumours. Hormone deprivation therapy is therefore used to inhibit further tumour growth, especially in patients with locally advanced or metastatic prostate cancer. Androgen deprivation can be achieved either by surgical or medical castration (see the current German interdisciplinary guideline for the early detection, diagnosis and therapy of the various stages of prostate cancer, September 2009 [2]). A medical standard therapy is the injection of synthetic analogues of gonadotropin-releasing hormone (GnRH; also known as luteinising hormone-releasing hormone, LHRH). These agents have a longer half-life and a higher affinity to the pituitary GnRH-receptor than physiological GnRH. After initial application of a GnRH-analogue, serum testosterone levels increase transiently over a period of one to two weeks. Testosterone inhibits the release of GnRH and the pituitary gonadotropin secretion via a negative feedback loop. Continuous administration of a GnRH analogue inhibits the pulsatile stimulation of gonadotropin secretion by physiological GnRH, which eventually leads to reduced androgen production. Within three weeks, most patients reach testosterone castration levels of < 20 ng/dL [3]. Leuprolide acetate, which is the active ingredient of Eligard^® ^45 mg (Astellas Pharma GmbH), is one of the most commonly used GnRH analogues.

Eligard^® ^(Astellas Pharma GmBH) is a new formulation of leuprolide acetate, which combines the active ingredient with a biodegradable polymer matrix (Atrigel^® ^delivery system) to achieve sustained release of leuprolide acetate. In Germany, the 1-month depot (Eligard^® ^7.5 mg) was approved in December 2003 and the 3-month depot (Eligard^® ^22.5 mg) was approved in January 2004 for the treatment of advanced hormone-dependent prostate cancer. The newest product, 6-monthly leuprolide acetate (Eligard^® ^45 mg), was approved for the German market in November 2006 and was launched in March 2007. The liquid drug is injected subcutaneously and forms a gel-like depot that slowly disintegrates and continuously releases the active ingredient. A 12-month, open-label, multicentre clinical trial [4] showed that in patients with prostate cancer, 6-monthly leuprolide acetate induced reliable and effective suppression of testosterone comparable to bilateral orchiectomy. Here we report the results of a non-interventional study with 6-monthly leuprolide acetate. Based on a broad patient population, this study investigated whether the efficacy and safety findings of the clinical study could be confirmed in daily clinical practice in a large number of patients (n = 1273). Experiences with the long dosing interval were of special interest in this study.

## Methods

The prospective, non-interventional study was performed in accordance with the Arzneimittelgesetz § 67 section 6 [5]. A total of 634 urologists who had decided to treat patients with advanced prostate cancer with 6-monthly leuprolide acetate participated in this study. Diagnosis and treatment decisions were at the sole discretion of the treating physicians. During the study period, which lasted from May 2007 to December 2008, study-related data from 1273 patients were captured. The observational period for each patient was 12 months. Concentrations of prostate-specific antigen (PSA) and serum testosterone (optional) were documented at the baseline visit and after 6 and 12 months. The physicians provided a global assessment of therapy and collected data on the local tolerability of 6-monthly leuprolide acetate. Physicians also reported on their experience of using the product and the handling of the syringe. In addition, the occurrence of adverse events was monitored and documented during the entire observational period. Data were processed in pseudonymised form and analysed descriptively.

The present study was conducted in accordance with the Declaration of Helsinki, and was approved by the Landesärztekammer Baden-Württemberg. All patients provided written, informed consent.

## Results and discussion

The mean age of the patients was 75 years. Further demographic and medical characteristics of the patients are summarised in Table 1. Approximately half of the patients (48%) were treated with leuprolide acetate for the first time. Of the remaining patients, one third were pre-treated with a different monthly depot formulation (either 1- month or 3- monthly) of the Astellas leuprolide acetate, and two thirds were pre-treated with other GnRH analogues. The majority of patients received GnRH analogue monotherapy, and 35.3% were given additional treatment. In most cases the additional treatment was anti-androgen therapy (78,9%, Table 2). A punch biopsy had been performed in 95% of the patients before the start of the study. The mean Gleason sum (2-10) was 6.8. Premature discontinuation of therapy was documented for 6% of the patients (n = 76). The most common reasons for discontinuation were loss to follow-up (26%, n = 20) and patient death during the study (22%, n = 17).

**Table 1:** Characteristics of documented male patients with advanced prostate cancer (total number = 1273)

Demographic data: mean (minimum, maximum)	
Age (years)	75 (50, 97)
Weight (kg)	81 (45, 160)
Height (cm)	175 (157, 198)
Diagnosis/findings: mean	

Time since first diagnosis (months)	33
Biopsy: Number of cores	8.2
Number of positive cores	4.5
cT1-2, %	44
cT3-4, %	32
M+, %	20
Gleason grade (1-5)	3.4
Gleason sum (2-10)	6.8

Therapy with GnRH analogues: number of patients (%)	

First-time treatment	610 (48)
Pre-treatment*	652 (51)
3 monthly leuprolide acetate	210 (32.2)
Leuprolide acetate, Takeda Pharmaceutical	168 (25.8)
Goserelin acetate, AstraZenca	107 (16.4)
Buserelin acetate, Sanofi-Aventis	97 (14.9)
1 monthly leuprolide acetate	27 (4.1)
Leuprorelin acetate, Takeda Pharmaceutical	21 (3.2)
Other drugs	22 (3.4)
Missing data	15 (2.3)

*Percentage refers to patients with change of therapy (n = 652). Total n = 667 includes multiple namings: single n = 637; 2-fold: n = 15

**Table 2:** Classification of patients receiving monotherapy or combination therapy

Therapy	Number of patients (%)
Monotherapy GnRH	724 (56.9)
Combination therapy	449 (35.3)
Bicalutamide	183 (40.8)*
Flutamide	171 (38.1)*
Zoledronic acid	59 (13.1)*
Cyproterone	57 (12.7)*
Oestramustine	12 (2.7)*
Docetaxel	6 (1.3)*
Missing data	10 (2.2)*
Total	498 (**)
Missing data	100 (7.9)
Total	1273 (100.0)

*Percentage refers to patients receiving a combination therapy (n = 449); **Total includes multiple naming for combination therapy: Single: 405; 2-fold: 40; 3-fold: 3; 4-fold: 1

### Effective reduction of PSA and testosterone levels

**Figure 1:**
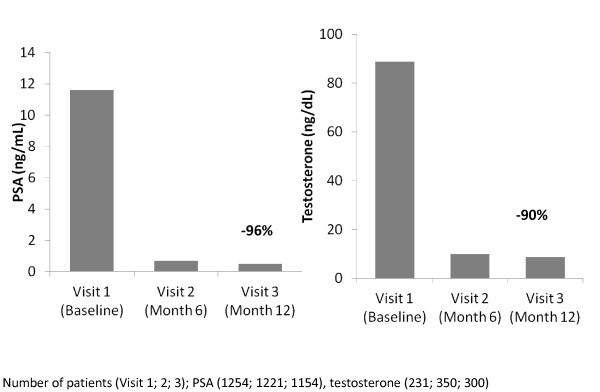
**Median serum concentrations of PSA and testosterone during treatment with 6-monthly leuprolide acetate**.

Within the first six months, a 94% decrease in the median PSA value from 11.6 ng/mL to 0.7 ng/mL was observed (Figure 1). PSA decreased further to a median value of 0.5 ng/mL at the end of the observational period (corresponding to a 96% decrease). At this time point, 50% of the patients had PSA values between 0.1 and 1.9 ng/mL (interquartile range). Measurement of serum testosterone was optional. Testosterone concentrations were available for up to 350 patients, i.e. up to 29% of patients with PSA measurements, at each visit (Figure 1). The median serum testosterone concentration decreased from 89 ng/dL to 10 ng/dL during the first six months, and decreased further to 9 ng/dL at the end of the observational period, corresponding to a 90% decrease in serum testosterone.

PSA and testosterone concentrations were also analysed in two subpopulations of patients who were pre-treated with other GnRH analogues (patients with complete data only). One of the subpopulations consisted of patients who switched from leuprolide acetate, Takeda Pharmaceutical (Trenantone^®^) to 6-monthly leuprolide acetate monotherapy (n = 99); the second subpopulation consisted of patients who switched from goserelin acetate, AstraZenca (Zoladex^®^) to 6-monthly leuprolide acetate monotherapy (n = 57). Within the first six months, a 93% decrease in the median PSA value was observed in the subpopulation that switched from leuprolide acetate, Takeda Pharmaceutical to leuprolide acetate, Astellas Pharma GmbH, from 5.8 ng/mL to 0.4 ng/mL. A 96.5% decrease in the median PSA value from 8.6 ng/mL to 0.3 ng/mL was observed in the subpopulation that switched from goserelin acetate to leuprolide acetate, Astellas Pharma GmbH. The median serum testosterone levels decreased from 150 ng/dL to 17.3 ng/dL during the first six months in the leuprolide acetate, Astellas Pharma GmbH subpopulation, corresponding to an 88% decrease. The median serum testosterone levels decreased from 72.3 ng/dL to 18.0 ng/dL during the first six months in the goserelin acetate subpopulation, and decreased further to 16.3 ng/dL at the end of the observational period, corresponding to a 77% decrease. These data demonstrate an effective reduction of PSA and testosterone levels by leuprolide acetate, Astellas Pharma GmbH in patients who either received a GnRH analogue for the first time or were switched from another GnRH analogue to leuprolide acetate, Astellas Pharma GmbH.

### Favourable therapy evaluation

**Figure 2:**
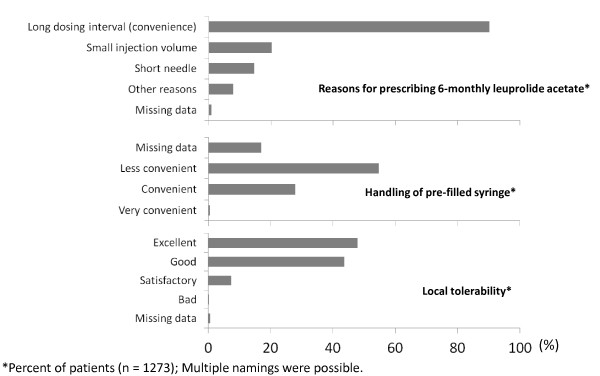
**Final assessment of 6-monthly leuprolide acetate therapy by the participating urologists**. **Legend**. Diagnosis and treatment decisions were at the sole discretion of the treating physicians. The physicians stated that the most frequent reasons for choosing 6-monthly leuprolide acetate were the long dosage interval, small injection volume, and short cannula. Less frequent reasons for choosing 6-monthly leuprolide acetate included compliance/patient's request, costs, physician's interest in 6-monthly leuprolide acetate, and good efficacy of the Astellas leuprolide acetate. Other reasons were named in minor frequencies.

The most frequent reason (90%) for prescribing 6-monthly leuprolide acetate, as given by the participating urologists, was the long dosing interval of the 6-months depot (Figure 2). Other common reasons were the small injection volume (20%) and the short needle of the pre-filled syringe (15%). A doctor's assistant prepared the syringe for more than half of the patients (57%), whereas physicians prepared the syringe in 42% of the cases (data was missing for 1% of patients). Handling of the pre-filled syringe was regarded as convenient or very convenient by the majority of users (72%; Figure 2).

### Good tolerability

The local tolerability of the product was assessed as good or very good for most patients (92%) (Figure 2). Adverse events (AEs) occurred in 108 (9%) of 1273 documented patients (Table 3). Non-serious AEs were reported for 69 patients (5%) and serious adverse events (SAEs) were documented in 39 patients (3%). No systematic allergic reactions were reported for any patient. For 17 of 69 patients, a causal relationship between the occurrence of a non-serious AE and the administration of 6-monthly leuprolide acetate was excluded, whereas 34 patients had AEs which were assessed by the physicians as definitely related to treatment. A causality assessment was missing or was deemed not possible for 18 patients with non-serious AEs. Most reported SAEs were tumour progression (i.e. metastasis) or the requirement of surgical measures including radical prostatectomy, surgery and hospitalisation. 25 patients (2%) died during the study. For 33 of 39 patients with SAEs, a causal relationship between the SAE and treatment with 6-monthly leuprolide acetate was excluded or assessed as being unlikely. For one SAE (tumour progression with fatal outcome) in an 82-year-old patient, a causal relationship to 6-monthly leuprolide acetate could not be excluded. A causality assessment was missing for 5 patients with SAEs.

**Table 3:** The most common non-serious adverse events

Adverse event	Number of patients (%)*
Local reaction at injection site:	41 (3.2)
Irritation	21 (1.6)
Nodule	7 (0.5)
Pain	5 (0.4)
Induration	3 (0.2)
Swelling	2 (0.2)
Burning sensation	2 (0.2)
Pruritus	1 (0.1)
Hot flushes	15 (1.2)

*Percentages are based on the total number of patients (n = 1273)

### Comparison to previous experiences with 6-monthly leuprolide acetate

In the above-mentioned clinical trial by Crawford and colleagues [4], 6-monthly leuprolide acetate achieved a 97% decrease in PSA. At the end of the study (Month 12), the mean testosterone concentration of 103 patients was 12.3 ng/dL (corresponding to 0.426 nmol/L), which is well below the castration level. The present study is the first non-interventional study performed since the launch of 6-monthly leuprolide acetate in Germany in 2007. The results of the study show that under the conditions of daily urological practice, PSA and testosterone levels could be decreased to a similar extent as in the clinical trial, confirming the therapeutic benefit of the 6-month depot. In the current investigation, the non-interventional study design provided a valuable tool, despite its known methodological limitations, for the evaluation of the efficacy and tolerability of a new formulation. This study was based on biochemical data that were extrapolated to be an attribute of patient improvement, which might not be realised for every patient in this non-interventional study. An additional limitation could be that interpretation of the data was influenced by approximately 50% of the patients who previously received androgen blockade therapy. In contrast to clinical trials, a larger and more heterogeneous group of patients could be included, and a broader range of physicians could be involved. Furthermore, this non-interventional study allowed the collection of data that was not accounted for in the Crawford clinical trial, such as data on the handling of the syringe and a global assessment of the therapy by the physicians. With regard to the tolerability data, the low incidence of documented hot flushes was notable. Results from clinical trials show that the occurrence of hot flushes is usually very common (> 1/10) [6]. A possible explanation for this discrepancy may be that many physicians rate the occurrence of hot flushes not as an AE, which needs to be documented, but rather as a 'normal' reaction under hormone deprivation, indicating the effectiveness of the GnRH therapy.

## Conclusion

The long dosing interval of 6-monthly leuprolide acetate was the main reason given by the participating urologists for making the prescribing decision with regard to the hormone deprivation therapy of their patients. In particular, physicians felt that patients with stable disease, patients who like to travel, or who have difficulty reaching a practice, may benefit from a longer interval between two injections. The present non-interventional study showed that the reliable reduction of PSA and testosterone levels achieved by 6-monthly leuprolide acetate injections twice a year, as demonstrated in the clinical study, can also be achieved under the conditions of daily urological practice. Furthermore, this study also confirmed the good tolerability of 6-monthly leuprolide acetate in routine clinical use.

## Conflicts of interest

UWT has received financial support for research projects and lectures from Abbott, Astellas, Lilly, Novartis, Sanofi-Aventis, and Takeda.

## Authors' contributions

UWT takes full responsibility for the manuscript.

## Pre-publication history

The pre-publication history for this paper can be accessed here:

http://www.biomedcentral.com/1471-2490/11/15/prepub
